# Formulating a novel drilling mud using bio-polymers, nanoparticles, and SDS and investigating its rheological behavior, interfacial tension, and formation damage

**DOI:** 10.1038/s41598-023-39257-5

**Published:** 2023-07-26

**Authors:** Ramin Taghdimi, Babak Kaffashi, Mohammad Reza Rasaei, Mohammad-Saber Dabiri, Abdolhossein Hemmati-Sarapardeh

**Affiliations:** 1 School of Chemical Engineering, College of Engineering, University of Tehran, Tehran, Iran; 2 Institute of Petroleum Engineering, School of Chemical Engineering, College of Engineering, University of Tehran, Tehran, Iran; 3 Department of Petroleum Engineering, Shahid Bahonar University of Kerman, Kerman, Iran; 4 State Key Laboratory of Petroleum Resources and Prospecting, China University of Petroleum (Beijing), Beijing, China

**Keywords:** Energy science and technology, Engineering

## Abstract

Formation damage is a well-known problem that occurs during the exploration and production phases of the upstream sector of the oil and gas industry. This study aimed to develop a new drilling mud formulation by utilizing eco-friendly bio-polymers, specifically Carboxymethyl Cellulose (CMC), along with nanostructured materials and a common surfactant, sodium dodecyl sulfate (SDS). The rheological properties of the drilling fluid and the impact of additives on its properties were investigated at the micromodel scale, using a flow rate of 20 mL/h. The polymer concentration and nano clay concentration were set at two levels: 0.5 wt% and 1 wt%, respectively, while the surfactant content was varied at three levels: 0.1 wt%, 0.4 wt%, and 0.8 wt%. The results of the interfacial tension (IFT) analysis demonstrated a significant decrease in the interfacial tension between oil and water with the increasing concentration of SDS. Furthermore, following the API standard, the rheological behavior of the drilling fluid, including the gel strength and thixotropic properties of the mud, was evaluated with respect to temperature changes, as this is crucial for ensuring the inherent rheological stability of the mud. The rheological analysis indicated that the viscosity of the mud formulation with nanoparticles experienced a reduction of up to 10 times with increasing shear rate, while other formulations exhibited a decline of 100 times. Notably, the rheological properties of the Agar specimen improved at 150 °F due to its complete solubility in water, whereas other formulations exhibited a greater drop in viscosity at this temperature. As the temperature increased, drilling fluid containing nanostructured materials exhibited higher viscosity.

## Introduction

Formation damage refers to the reduction in absolute permeability or the decrease in the relative permeability of the production fluid. This damage can be minimized by using appropriate drilling fluids. Different types of drilling fluids, including oil-based drilling mud (OBDM), water-based drilling mud (WBDM), gas-based drilling mud (GBDM), and their respective additives, are used in combination with suitable clay additives^1^.

The choice of the base drilling fluid significantly influences its behavior during drilling operations. OBDM is widely recognized as the superior system due to its lubricating properties^2^. This system provides wellbore stability, low torque and drag, excellent fluid loss control and filter cake quality, appropriate rheological properties for hole cleaning, and temperature stability^3,4^. Shale formations tend to swell when infiltrated by water-based drilling fluids (WBDF), leading to instability of the well wall. To prevent swelling, OBDM is often preferred since there is no interaction between oil and shale. However, in order to address environmental concerns, water-based muds (WBM) can also be utilized. Thus, drilling fluids play a crucial role in the oil and gas industry^5,6^.

Drilling fluids serve numerous functions in drilling operations, including well cleaning, formation pressure control, cuttings suspension, sealing permeable formations, wellbore stability, reduction of formation damage, cooling, lubrication, support for the bit and drilling assembly, hydraulic energy transfer to tools and bit, ensuring adequate formation evaluation, corrosion inhibition, environmental impact reduction, and simplifying cementing and completion drilling mud^7–9^. These drilling fluid functions are achieved through the use of complex chemistry based on additives. Additives are used to enhance the properties of drilling fluids, such as density, rheology, fluid loss, alkalinity, salt content, solid content, oil–water ratio, sand content, electrical stability, and other relevant properties. The density of the drilling fluid is particularly important, as excessive increases can lead to formation failure^10,11^. From Rheological properties, including apparent viscosity (AP), plastic viscosity (PV), and yield point (YP), are crucial characteristics during drilling operations^12,13^. Among the various rheological properties, gel strength (GS) is defined as the mud’s ability to keep mud particles suspended^14,15^. The strength property of the mud plays a key role in drilling horizontal and multi-branch wells.

Loss of the gelatinous property can cause suspended particles in the drilling fluid to settle, leading to accumulation around the drill bit and sticking in the drill string. In recent years, there have been advancements in nanomaterials technologies, and researchers have explored their application in the petroleum industry^16–19^. The investigations result showed that nanoparticles can be used as suitable additives to improve drilling fluid properties^20–22^. The unique mechanical, hydrodynamic, thermal, electrical, chemical properties, and interaction potential of nanoparticles make them an excellent choice for drilling fluid applications^22–24^.

Jung et al.^25^ observed that the addition of Fe_2_O_3_ nanoparticles with a particle size ranging from 3 to 30 nm in WBDM at a concentration of 5 wt% of nanoparticles improves the rheology of the drilling fluid. Barry et al.^26^ used Fe_2_O_3_-clay hybrid nanoparticles with sizes of 3 and 30 nm in WBM containing 5 wt% bentonite. They observed that the addition of these nanoparticles enhances the rheological performance of the drilling fluid. Wang et al.^27^ employed nanoparticles with sizes of 10–20 nm in WBDF with 4 wt% bentonite, and concluded that an optimal concentration range between 0.05 and 0.5% by weight leads to improved rheology, filtration properties, and thermal properties of the drilling fluid. Javeri et al.^28^ utilized SiO_2_ nanoparticles with a particle size of 50 nm at concentrations of 5 wt% in WBDF. They found that these additives reduce the thickness of the mud cake. Cheraghian et al.^29^ investigated the use of nano silica in 5% by weight of WBDF and observed its effect on the rheology of the drilling mud. Agarwal et al.^30^ studied the use of nano clay and nano silica in high-pressure high-temperature (HPHT) invert emulsion-based drilling fluids and their impact on the rheology of the drilling mud. Abdo and Haneef^31^ employed nano clay with a particle size between 10 and 20 nm in WBDF with bentonite and discovered that the addition of nano clay controls viscosity under high temperature and pressure conditions. Cheraghian^29^ also concluded that the addition of nano clay has a positive effect on the rheology and filtration properties of WBDF.

Sadeghalvaad and Sabbaghi^32^ successfully synthesized a polymer-nanocomposite (TiO_2_/PAM) for use in the WBDF system. Their results demonstrated that the nanocomposite improved rheological properties, as well as the volume of fluid loss and thickness of mud cake. Aftab et al.^33^ synthesized and characterized a ZnO-Acrylamide composite, which modified the chemical and thermal properties of drilling fluids. The composite exhibited modifications in filtrate loss, lubricity, yield point, and gel strength, making it a suitable additive for modifying shale swelling. Huang et al.^34^ investigated the effects of a nanocomposite of SiO2/acrylic resin with a core–shell structure on the rheological characteristics and thermal properties of WBDM. They found that drilling fluids with acrylic/SiO_2_ nanoparticles increased plugging efficiency and reduced fluid invasion.

In another study, Khani et al.^35^ used a new drilling fluid with clay nanoparticles to improve thermal conductivity. They concluded that the presence of nano clay in the drilling fluid enhances thermal stability. William et al.^36^ evaluated the use of CuO and ZnO nanoparticles in 0.4 wt% XG in WBDF to improve thermal properties under high-pressure/high-temperature (HP/HT) conditions. Wang et al.^37^ focused on the mechanical performance of drilling fluids designed with silica nanoparticles for use in natural gas hydrate exploration. They observed that the use of hydrophilic silica nanoparticles in drilling fluids resulted in 10% less hydrate formation compared to ultrapure water.

Perveeen et al.^20^ conducted a study on the effect of zinc titanate nanoparticles on the rheological and filtration properties of WBDF. They discovered that the use of nanoparticles in WBM improved rheological properties, and the addition of electro spun ZnTiO at a concentration of 0.3 w/v% reduced fluid loss by almost 30%. Kafashi et al.^23^ conducted a study on the rheological properties and the possibility of nano (Na, Ca)-bentonites nanoproducts to meet the required drilling mud properties. According to the results obtained from flow properties tests for the mixture, it indicated that the mixture was not adequate to be a suitable drilling fluid. In another study, Kakashi et al.^24^ investigated nanoclay absorbents and additives’ effects on modification of rheological properties of drilling fluids in porous media using glass micromodel. Their results showed that 75 wt% of nanoclay suspension and polyanionic cellulose (PAC) could successfully improve the rheological properties. Later, Alkalbani et al.^38^ evaluated the use of SiO_2_ nanoparticles in WBM to improve the rheological properties of drilling fluid in deep reservoirs. Their results indicated that the silica nanoparticles improved the rheological properties and produced greater viscosity and yield point by more than 50%. Recently, Mikhienkova et al.^39^ evaluated the use of SiO_2_ nanoparticles in OBM to enhance the rheological properties of drilling fluid. They found that the addition of SiO_2_ nanoparticles resulted in a reduction of filtration losses in oil-based drilling fluids by 50–70%. Additionally, the inclusion of these nanoparticles significantly increased the colloidal stability of oil-based drilling fluids.

The presented work proposes a novel approach to formulating drilling fluid by utilizing a combination of carboxymethyl cellulose (CMC), eco-friendly Agar polymer, nanostructure material, and typical surfactants. Subsequently, the rheological properties of the drilling fluid and the impact of additives are investigated using the micromodel scale to achieve rheological stability. The innovation of this study lies in the utilization of biocompatible agar polymer in drilling mud and the exploration of agar polymer compounds combined with nano clay and CMC to effectively control the thickness of the mud cake. CMC, which serves as a base polymer in general drilling mud formulations, is employed in this study. Furthermore, the addition of nanoclay to the drilling mud formulation and its injection into a glass micromodel enables the precise measurement and reporting of the mud cake’s exact quantity. Furthermore, the introduction of nanoclay and biocompatible agar polymer into the formulation allows the measurement and reporting of the flower cake amount for the first time in a glass micromodel. The rheological behavior of the polymer nanocomposite and the quantities of key indicators are examined and presented. One of the advantages of employing nanoclay over other nanoparticle structures is its cost-effectiveness, performance, and enhanced compatibility with bentonite. Additionally, agar, being a bio-ecological polymer indigenous to Iran, offers substantial added value compared to other polymers.

## Materials and methods

### Materials

In this work, all materials used were commercial grades obtained from a petrochemical company. Bentonite with a particle size of 200 mesh and Montmorillonite Cloisite 30B with a surface spacing of 1–5 nm were purchased from the National Iranian Oil Company. The properties of the oil and sodium dodecyl sulfate (SDS) are presented in Tables 1 and 2, respectively.

**Table 1:** Extracted oil from the Asmari oil reservoir (obtained from Tehran Petrochemical Company).

Density (kg/m^3^)	Salt quantities (wt%)	Total acid number (mg KOH/mg)	Sulfur quantities (wt%)	Mercaptan (ppm)
863.5	8	0.25	1.64	44.5

**Table 2:** Characteristics of SDS surfactant.

Chemical	Critical Micelle concentration (mol/L)	Molecular weight (g/mol)	Concentration (wt)
SDS	8 × 10^–3^	288.372	0.1, 0.4, 0.8

Carboxymethylcellulose sodium salt with a medium molecular weight was obtained from PRS Panreac Química SAU (Barcelona), and Agar bacteriological dehydrated, suitable for laboratory applications, was provided by Quelab/UK. Agar is insoluble in cold water but exhibits significant swelling properties. It has the ability to absorb water up to 20 times its own weight. Agar is soluble in water at 80 °C and can form a stable gel at concentrations below 0.5% by weight.

### Methodology

Due to limitations in the number of experiments, the experimental procedures were conducted using the D-Optimal method proposed by Minitab software. Four factors were considered: flow rate, polymer concentration, surfactant content, and nano clay concentration. The penetration of drilling fluid into the micromodel was recorded six times. The experiments were classified to assess the penetration of drilling fluid into the micromodel. The following parameters were studied: a flow rate of 20 mL/h for the micromodel, polymer and nano clay concentrations at two levels (0.5% and 1% by weight), and surfactant content at three levels (0.1%, 0.4%, and 0.8% by weight).

The drilling fluid was prepared following API standards. The procedure involved weighing 10.5 mg of bentonite and adding it to 350 mL of water. The mixture was stirred using a WiseStir overhead stirrer at 25 °C and a stirrer speed of 250 rpm for 2 h. The mixing process was then conducted in an ultrasonic bath, specifically utilizing the Elma Ultrasonic S80H from the USA. A base frequency of 40 kHz was employed, and the process was repeated three times, with each iteration lasting 15 min. The objective of this approach was to enhance the stability of the suspension. After achieving homogeneity, the mixing process was stopped and the temperature was controlled. The rheological properties of the drilling fluid were determined using an MCR300 rheometer at ambient temperature. The interfacial tension (IFT) between oil–water systems, in terms of SDS content in the aqueous solution, was measured using the pendant drop method. Finally, the drilling fluid was injected into the micromodel, and the behavior of the two-phase fluid in the porous media was observed using an optical microscope. It is important to note that all experiments on the drilling fluid formulation followed the same procedure.

The rheological behavior of the drilling fluid was measured using an MCR300 rheometer from Anton Paar, Graz, Austria. The experiments followed the rheological test procedure for drilling fluids. The required time to reach a steady state was investigated at ambient temperature. Subsequently, the fluid loss was measured using a filter press low-pressure low-temperature (LPLT) instrument. A pressure of 100 psi was applied, and the amount of filtrate discharged in 30 min was measured. The water-bentonite suspension (base mud) was injected into the micromodel, and the penetration of fluid into the porous media was measured by continuously taking pictures. The thickness of the mud cake was assessed under a microscope at a flow rate of 1 mL/h after 50 min of injection. The same experimental method was employed for all drilling fluid formulations. It should be mentioned that all experiments were conducted three times and the average values were reported. The uncertainty in IFT, particle size, and viscosity were ± 3%, 7%, and 5%, respectively.

A glass micromodel consists of a flow network etched onto the surface of a glass plate. There are two main categories of patterns: geometric network patterns and rock-look-alike patterns. In this study, a geometric network pattern representing a porous media was prepared using computer software, specifically Corel Draw. The porous media model was designed with real dimensions, including the size of pore throats and pores, using Corel Draw software and applied to the glass surface^40^.

## Results and discussion

### Particle size analysis

**Figure 1:**
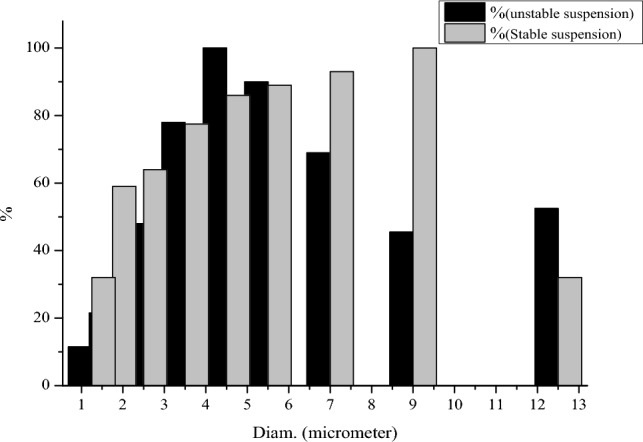
Differential reduction of bentonite particle diameter in stable state compared to unstable state in suspension using the centrifuge method.

The particle size was measured using an SA-CP3 particle size analyzer from Shimadzu, Kyoto, Japan. Figure 1 illustrates the reduction of bentonite particle size from a stable to an unstable state. The swelling properties of bentonite, which indicate viscosity alteration, were measured as the main parameter^41^. The addition of materials to the formulation depends on their swelling properties, which lead to changes in viscosity. Particle size analysis was conducted using both the simultaneous gravity and centrifuge methods, with a centrifuge speed of 500 RPM. The average diameter of bentonite particles in stable and unstable suspensions was found to be 8.34 µm and 4.72 µm, respectively. The increase in average particle diameter from a stable to an unstable state in the prepared suspension demonstrates the swelling behavior of bentonite.

**Figure 2:**
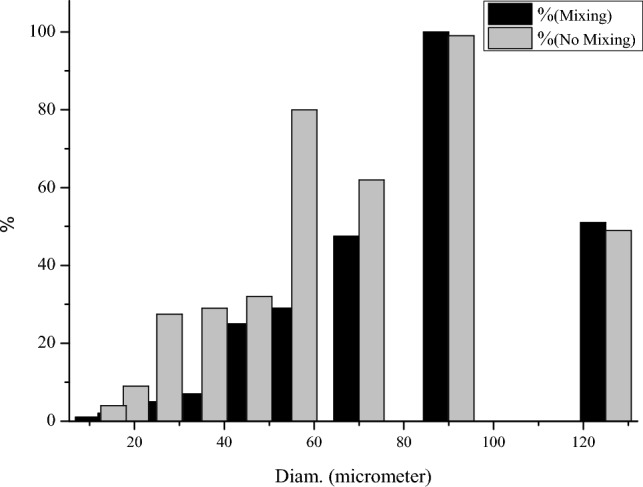
Differential Agar particle size with and without mixing process in water using the gravity method (1% wt of Agar).

Regarding Agar, the prepared suspension is insoluble at ambient temperature but swells twenty times in water at this temperature. The mesh size of Agar in the solid phase is 150 µm. Therefore, 1% by weight of Agar was added to 100 mL of water to determine the extent of its swelling. Two scenarios were studied to evaluate the increase in average diameter in the mixed and unmixed conditions (Fig. 2). The average diameter of Agar particles with and without mixing processes was measured to be 87.07 µm and 69.60 µm, respectively (the mixing time of Agar in water was set at 20 min). During the mixing process, the swelling value increased, directly affecting the fluid loss of the drilling mud.

### Interfacial tension measurement

**Figure 3:**
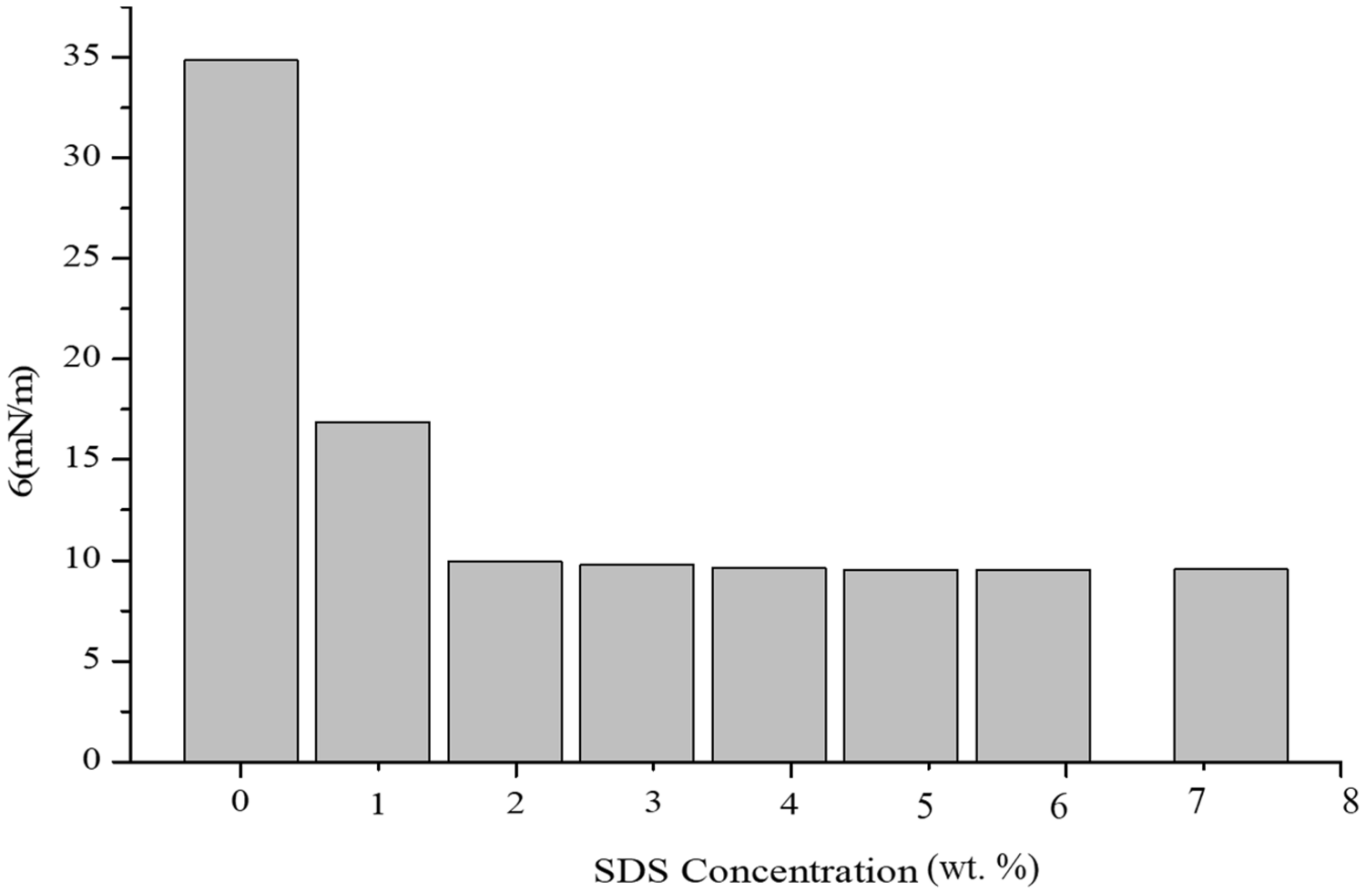
Interfacial tension (IFT) of the oil–water system.

The interfacial tension (IFT) between the oil–water system was determined by calculating it using the pendant drop method^42^. Figure 3 illustrates the variation in IFT with increasing SDS concentration. The tests were conducted at seven different levels corresponding to the critical concentration of the SDS surfactant. The results indicate that the IFT value remains constant at concentrations above 1 wt% of SDS. The IFT tests demonstrate that with the addition of varying amounts of SDS, the IFT between oil and water decreases significantly and reaches a plateau value of 9.55 mN/m.

**Figure 4:**
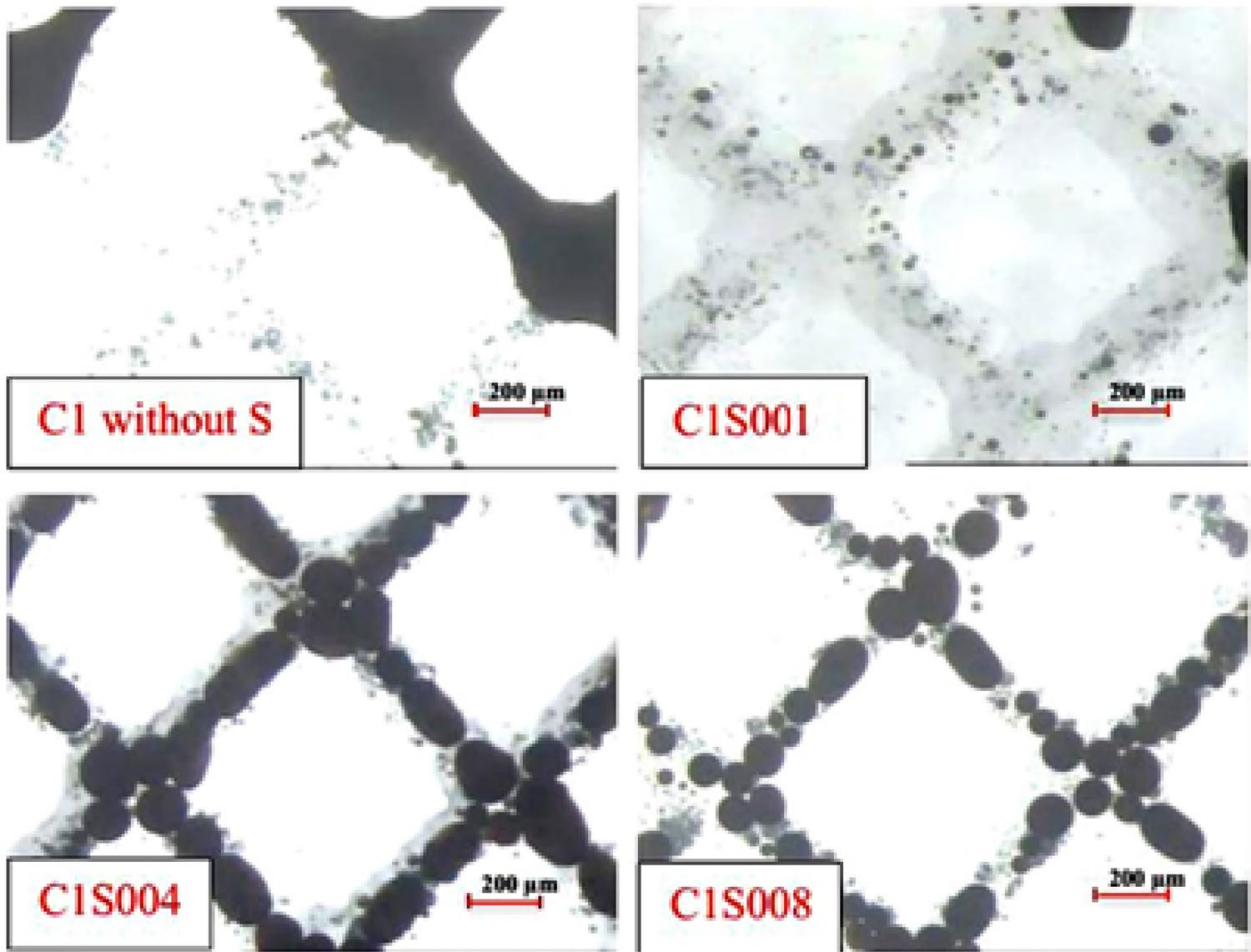
Effect of surfactant on oil droplet size in porous media.

The addition of surfactant significantly decreases the interfacial tension, facilitating the movement of oil and water in porous media. This leads to an expansion of the invaded zone, as depicted in Fig. 4. However, it is important to note that the addition of surfactant also causes an increase in the invaded zone, resulting in water blocking in formations containing mineral clay. The reduction in water blocking, which is achieved by mitigating drilling fluid attack and mineral clay swelling, is more cost-effective and beneficial than solely relying on surfactant addition. Therefore, incorporating surfactant into the drilling fluid is reasonable for formations that include mineral clay.

In the glass micromodel, where mineral clay is absent, the water-blocking phenomenon and the consequent reduction in absolute permeability, which are typically addressed by using surfactants, do not occur. Therefore, the application of surfactant in this context could have adverse effects, leading to an increase in the invaded zone. Figure 4 demonstrates the effect of surfactant on the oil particle diameter inside the micromodel for CMC fluid.

### Experimental rheometer

The rheological behavior of the drilling fluid was analyzed using an Anton Paar MCR 300 rheometer (Graz, Austria). Various rheological measurements, including the determination of gel strength, flow curve analysis, and assessment of fluid stability, were performed at ambient temperature. The objective of these rheological measurements was to identify the optimal drilling mud formulation by considering factors such as gel strength and the modification of thixotropic properties of the drilling fluid^24,43^.

**Figure 5:**
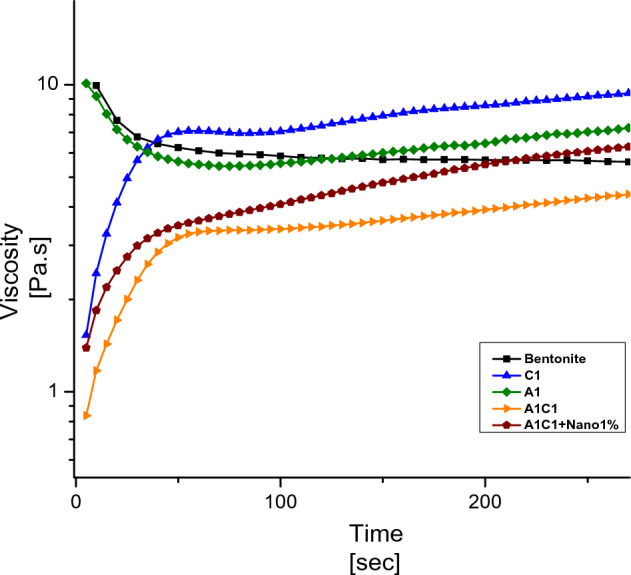
Plot of drilling fluid viscosity versus time at ambient temperature.

The time stability of the drilling fluid was assessed by plotting the viscosity against time at various shear rates ranging from 0.01 to 100 s^−1^ at ambient temperature. Figure 5 illustrates the time stability of the drilling fluid at ambient temperature. It can be observed that the addition of nanoparticles to the formulation enhances the viscosity of the fluid over time. In contrast, the fluid containing water and bentonite exhibits a decreasing trend in viscosity at ambient temperature^44^.

However, the base mud, which lacks the polymer additives that improve fluid properties, also shows a decreasing trend in viscosity. On the other hand, drilling muds containing Carboxymethyl Cellulose or Agar demonstrate better stability with increasing time at different shear rates. The drilling fluid that exhibits the highest stability at different time intervals consists of 1% wt of Agar, 1% wt of Carboxymethyl Cellulose, and 1% wt of Nano clay. The diagram clearly demonstrates the increased stability of the A1C1 + Nano 1% drilling fluid over time.

**Figure 6:**
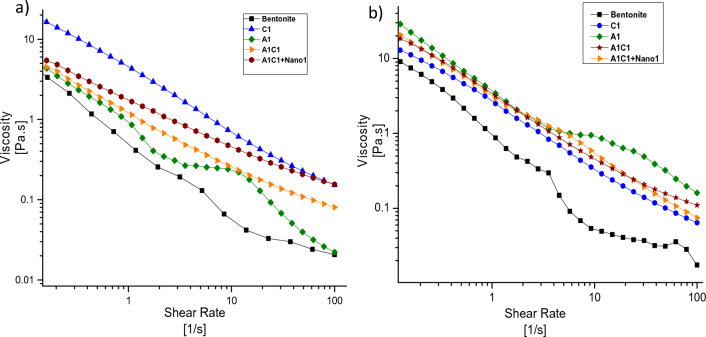
Variation of viscosity in terms of shear rate at (**a**) 120 and (**b**) 150 °F.

Figure 6 presents the viscosity behavior of the drilling fluid as the shear rate increases. It can be observed that fluids containing nanoparticles exhibit the least reduction in viscosity, indicating high stability with increasing shear rate. On the other hand, the base fluid shows the least stability. The presence of Agar alone does not significantly improve the rheological properties of the drilling fluid. However, when Agar is combined with carboxymethylcellulose, a synergistic effect occurs, leading to improved rheological properties and appropriate behavior at 120 °F.

The behavior of the drilling fluid containing Agar at the lowest and highest shear rates is similar to that of the base fluid at 120 °F. While the fluid containing Carboxymethyl Cellulose has the highest viscosity at the lowest shear rate, its viscosity drop is greater than that of the A1C1 drilling fluid as the shear rate increases at 120 °F.

However, the addition of Agar helps reduce the drop in viscosity. In the case of the A1C1 + Nano 1% wt drilling fluid, the viscosity drop is the lowest compared to other fluids as the shear rate increases. Therefore, the stability of the A1C1 + Nano 1% wt drilling fluid is higher than that of all other fluids in terms of shear rate at 120 °F. It is noteworthy that for other formulations, the viscosity drop is more significant at 150 °F compared to 120 °F. The viscosity drop in formulations containing nanoparticles at 150 °F is 6–8 times higher compared to that at 120 °F. At low shear rates, the nano clay acts as a lubricant, facilitating the movement of bentonite particles. However, at high shear rates, the nano clay forms a structure, leading to a drop in viscosity.

Furthermore, it is mentioned that the rheological properties of the Agar specimen alone were improved at 150 °F due to its complete solubility in water. However, in other formulations, a higher drop in viscosity is observed at this temperature. The presence of nanoparticles in the formulation causes a more significant viscosity drop at 150 °F compared to 120 °F, approximately 6–8 times higher.

At low shear rates, the presence of nano clay in the drilling fluid acts as a lubricant, promoting easy movement of the bentonite particles. This lubricating effect helps maintain the viscosity of the fluid at lower shear rates, allowing for better fluid flow and stability.

However, as the shear rate increases, the nano clay particles start to form a structure within the fluid. This structure formation leads to a drop in viscosity at high shear rates. The interactions between the nano clay particles and the surrounding fluid matrix result in a reduction in the resistance to flow, causing the viscosity of the fluid to decrease.

This behavior is commonly observed in shear-thinning fluids, where the viscosity decreases as the shear rate increases. The presence of nano clay in the drilling fluid can alter the flow behavior and rheological properties, providing both lubricating and structuring effects depending on the shear rate applied.

**Figure 7:**
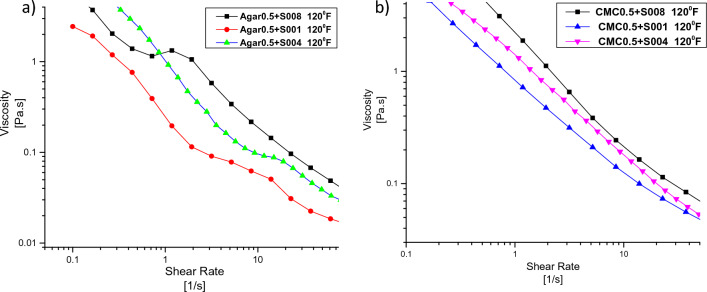
Effect of surfactant on viscosity at 120 °F: (**a**) drilling fluid containing agar, (**b**) drilling fluid containing CMC.

The presence of SDS surfactant in the drilling fluid formulation has several effects on viscosity. Firstly, the surfactant has an anionic nature, which can reduce water blocking in clay formations. This leads to an increase in the viscosity of the drilling fluid as the surfactant concentration is raised. The anionic surfactant interacts with the clay particles, promoting their swelling in water and consequently increasing the viscosity of the suspension. Furthermore, both Agar and carboxymethylcellulose are ionic compounds, and the presence of anionic surfactant enhances the interaction between these polymers and the surfactant, resulting in changes in viscosity. Figure 7 provides information on the effect of surfactant concentration on viscosity alteration for Agar and carboxymethylcellulose at a temperature of 120 °F.

It is worth noting that the viscosity changes associated with low surfactant concentrations are relatively small. The thixotropy measurements were also conducted to examine the recovery of the fluid’s structure after the application of high shear. Thixotropy refers to the phenomenon where a fluid’s viscosity decreases under shear stress but recovers over time when the shear stress is removed. These measurements provide insights into the structural behavior and stability of the drilling fluid under different shear conditions.

**Figure 8:**
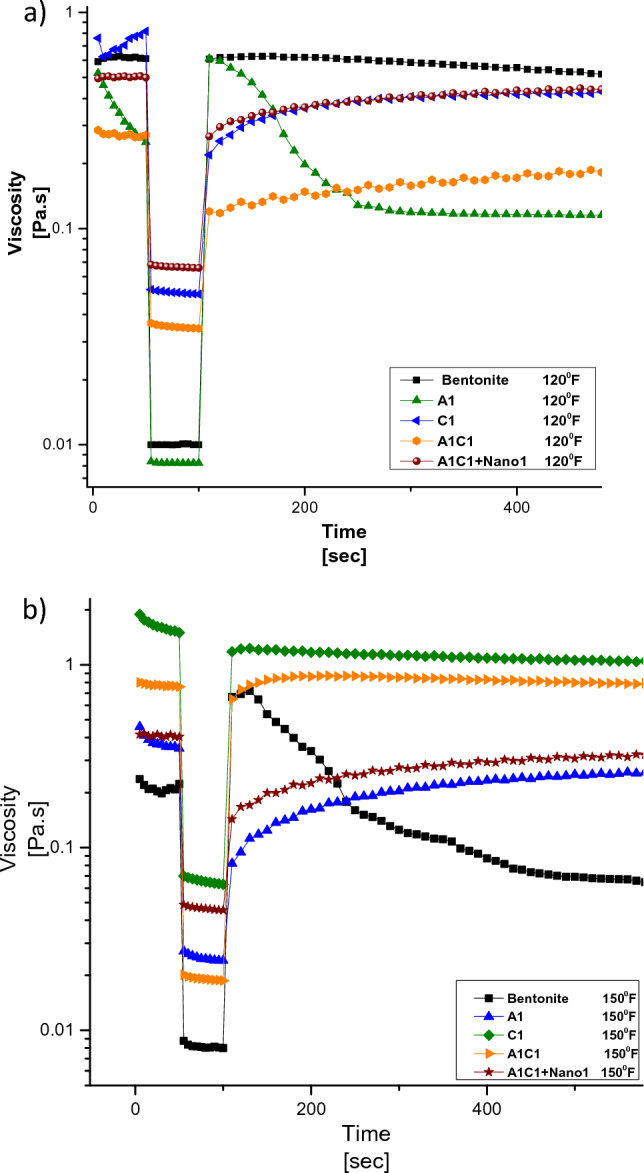
Thixotropic behavior of drilling fluid: (**a**) At 120 °F, (**b**) At 150 °F.

The thixotropy test is conducted to determine the time required for the drilling fluid to regain its initial viscosity after being subjected to a shear rate of 1022 s^−1^ (Fig. 8)^38,45–47^. The addition of Agar to the fluid results in a negative slope in the viscosity-time relationship during the third stage of the thixotropy test. This indicates that Agar exhibits a larger viscosity drop after surpassing the shear rate of 1022 s^−1^, potentially due to the collapse of intermolecular structures that were previously formed.

One advantage of Agar compared to CMC is that Agar has a viscosity value that is 10 times higher than CMC at the beginning of the third stage of the applied shear rate. This higher viscosity prevents Agar from immediately entering the porous media, allowing Agar and other additives to bridge the porous media.

The viscosity reduction after a rotation of 600 rpm induces thixotropic characteristics in the drilling fluid^23,24,48^. This behavior is observed for the fluid containing CMC, indicating anti-thixotropic properties. The anti-thixotropic behavior leads to an increase in viscosity and a decrease in permeability values of the porous media. On the other hand, the simultaneous increase in viscosity of Agar exhibits a restraining effect on permeability. CMC, with its gradual viscosity growth, slows down permeability. The combination of Agar and CMC results in rheopectic behavior.

Furthermore, the viscosity drop for the rotation rate of 600 rpm in the presence of nanoparticles is smaller compared to other formulations. This suggests that the presence of nanoparticles contributes to the stability of the drilling fluid’s thixotropic behavior.

At a temperature of 150 °F, the thixotropic behavior of the drilling fluid indicates that the Agar polymer remains stable as the temperature increases. Conversely, the base mud exhibits high instability at 150 °F. The dissolution of the Agar polymer at a shear stress of 1022 s significantly improves the properties of fluids containing Agar at 150 °F, as demonstrated in Fig. 8.

**Figure 9:**
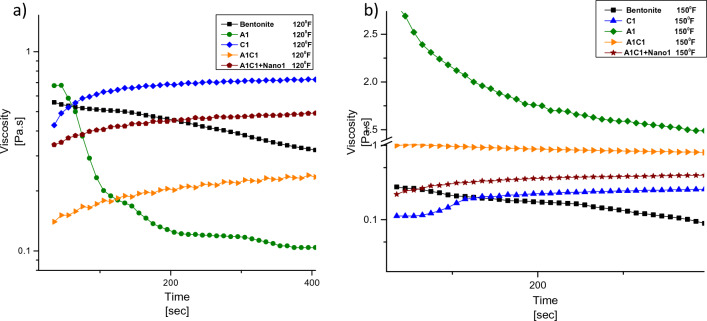
Gel strength of drilling fluid (**a**) at 120 °F, (**b**) at 150 °F.

In Fig. 9, the gel strength of the drilling fluid is illustrated. At a temperature of 150 °F, the dissolution of Agar in water leads to a significant increase in viscosity, reaching approximately 10 orders of magnitude higher compared to 120 °F. The base mud (bentonite fluid) exhibits an unstable behavior at this elevated temperature. Unlike the behavior observed at 120 °F, the viscosity drop is amplified by 10 times, and the gel strength of CMC is compensated by the presence of Agar in the A1C1 formulation.

Furthermore, an increase in the concentration of nanofillers contributes to an improvement in the gel strength of the drilling fluid. This indicates that the addition of nanofillers enhances the ability of the fluid to form a stable gel structure, potentially resulting in improved performance during drilling operations.

### Mud cake thickness and volume

**Figure 10:**
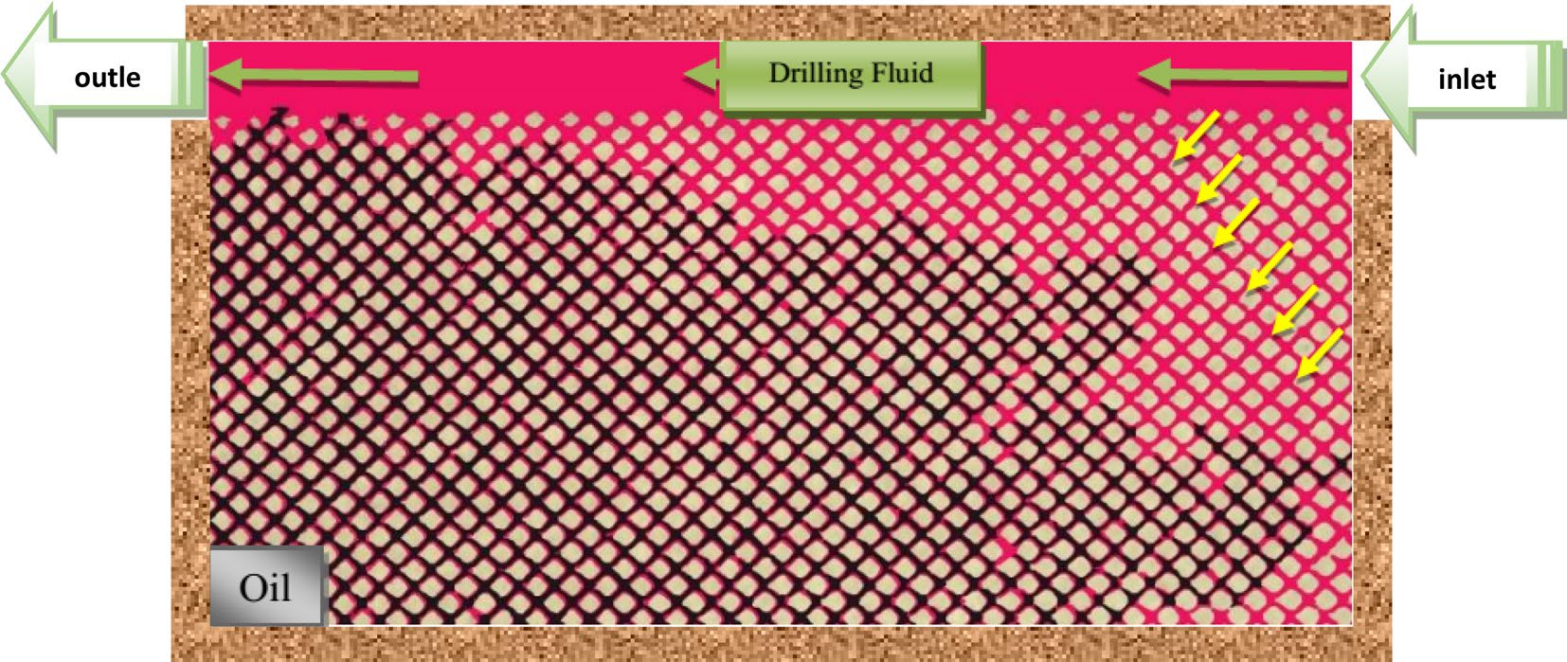
Bentonite injection and penetration of mud filtrate inside the micromodel.

The thickness of the mud cake was calculated by continuously capturing microscope images along the wellbore after 50 min of injecting the proposed mud formulation into the micromodel. The Fig. 10 illustrates the drilling fluid injection process and the resulting mud cake formed on the wellbore of the micromodel.

**Figure 11:**
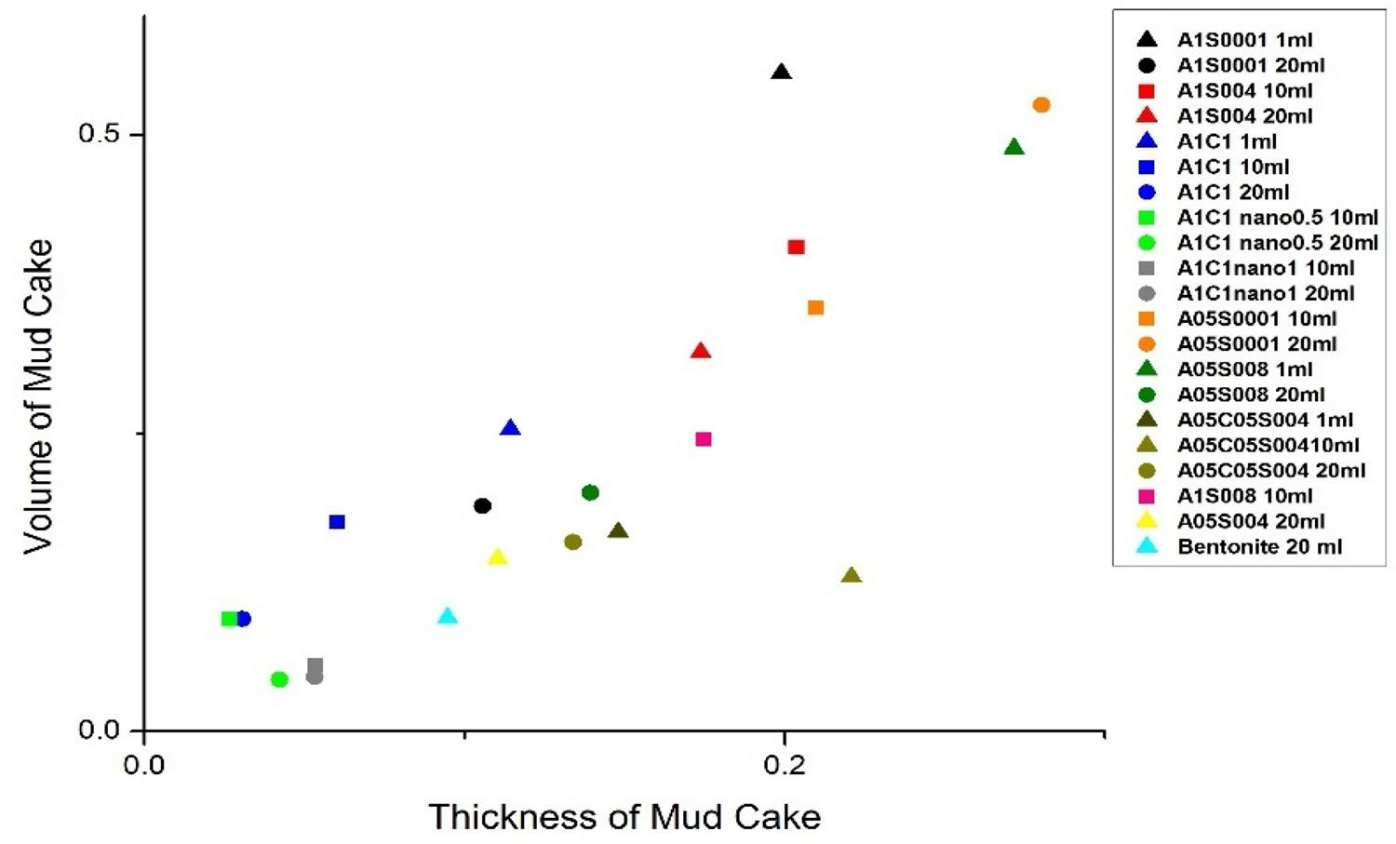
Normalized volume of mud cake versus thickness.

**Figure 12:**
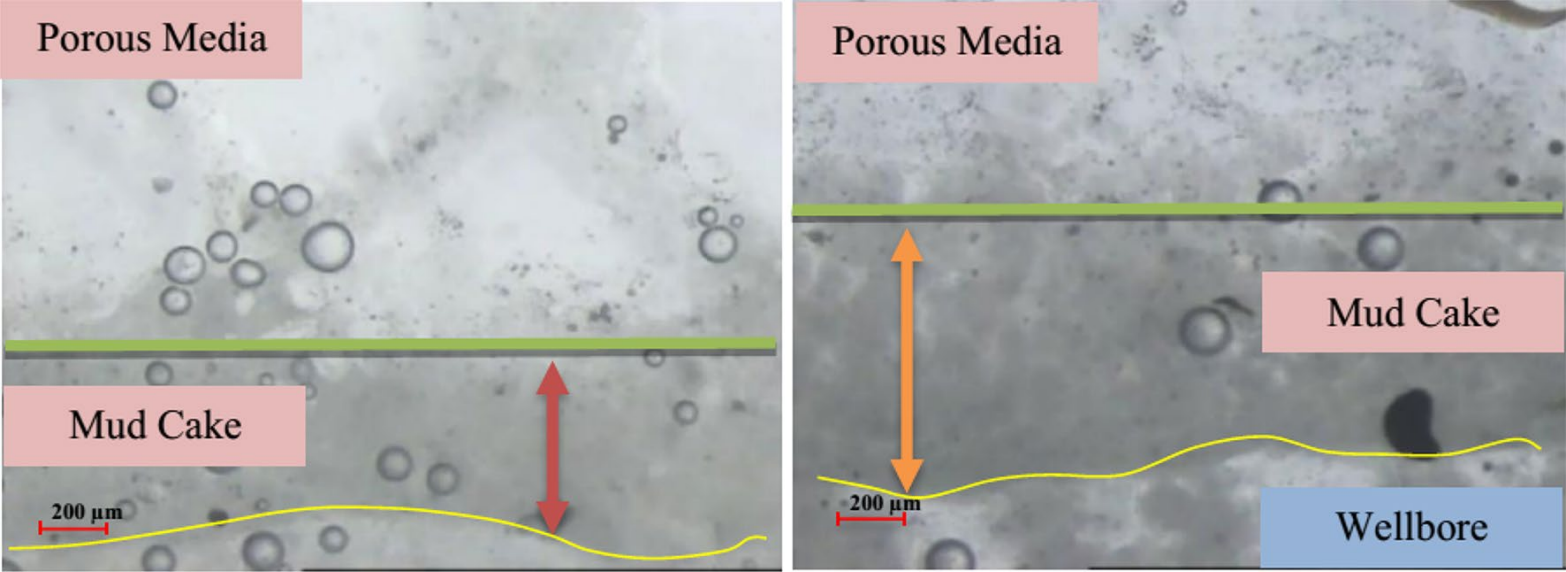
Formed mud cake at the main runner with a real size of 2 × 3 mm^2^.

The normalized results of mud cake thickness and volume on the micromodel wall are presented in Fig. 11. It is evident that the formulation containing CMC polymer fails to generate a mud cake on the wall, resulting in complete fluid penetration into the porous media and significant formation damage. Our objective was to achieve an optimal mud cake thickness with minimal penetration. Among the different formulations, A1C1 + nano1 wt% exhibited the least penetration. Figure 12 also demonstrates that as the flow rate increases, both the volume and thickness of the mud cake decrease, indicating that higher flow rates disrupt the integrity of the mud cake.

Filtration occurs in both dynamic and static conditions during the drilling process. Dynamic filtration occurs when the drilling fluid circulates, while static filtration occurs during periods of inactivity such as when joints are made or during trips when the fluid does not circulate^49^. In this section, we will focus on the study of dynamic filtration under atmospheric pressure and ambient temperature. Figure 12 demonstrates that the formation of a mud cake helps reduce fluid loss in non-reservoir permeable formations and prevents mud penetration into reservoir formations.

The homogeneity of the formed mud cake is crucial as it enhances wellbore stability and acts as reinforcement for weak formations. The size of the bridging agent should be approximately half the size of the largest pore throat. Agar polymer performs well as a bridging agent in this regard. It allows the formation of a filter cake at a temperature lower than its dissolution temperature. The swelling of the polymer at 120 °F contributes to the formation of the filter cake, which effectively blocks the penetration channels and prevents drilling mud from entering the micromodel. It is important to note that the drilling mud penetration test was conducted only at a temperature of 120 °F due to laboratory limitations.

On the other hand, the fluid containing CMC does not serve as an effective bridging agent due to its rheological properties, and it is unable to block the entry of the porous media.

Future studies could delve into the effects of varying temperature conditions on the observed phenomena. Conducting experiments at different temperatures can help identify temperature-dependent trends, such as changes in fluid flow behavior, chemical reactions, or interfacial phenomena. By investigating a range of temperatures, a more comprehensive understanding of the system’s response and potential temperature-induced limitations can be gained.

Moreover, exploring alternative patterns of micromodels can offer additional insights. Different micromodel patterns, such as heterogeneous pore structures or complex network geometries, may better mimic specific reservoir conditions or flow scenarios. This can help researchers evaluate the influence of pore-scale heterogeneity on fluid flow patterns, dispersion, and other relevant processes.

Furthermore, the utilization of three-dimensional micromodels presents a promising avenue for future research. Traditional two-dimensional micromodels provide valuable insights into fluid behavior, but they may oversimplify the complex nature of porous media. Employing three-dimensional micromodels can capture more realistic pore-scale geometries and connectivity, allowing for a more accurate representation of flow processes. This advancement can help address the limitations of two-dimensional models and provide a better understanding of flow dynamics, multiphase behavior, and transport phenomena.

By expanding investigations to encompass these aspects, future studies can significantly contribute to the field of micromodel-based research, enhancing our understanding of fluid flow in porous media and aiding the development of more efficient and accurate reservoir engineering practices.

## Conclusions

In this study, the rheological properties of the drilling fluid and the impact of additives were investigated using a micromodel scale with a flow rate of 20 mL/h. The concentration of polymer and nano clay was studied at two levels (0.5 wt% and 1 wt%), and the surfactant content was studied at three levels (0.1 wt%, 0.4 wt%, and 0.8 wt%). Additionally, the interfacial tension (IFT) between oil–water systems was measured using the pendant drop method, focusing on the SDS content in the aqueous solution. The following conclusion could be drawn from this study.

The results of the IFT analysis revealed a significant decrease in interfacial tension between oil and water with an increase in SDS concentration. The rheological results demonstrated that fluids containing nanoparticles exhibited the highest stability as the shear rate increased. The complete dissolution of Agar in water resulted in improved rheological properties at 150 °F. The viscosity drops observed at 150 °F were approximately 5–10 times higher than those at 120 °F. With an increase in the concentration of the anionic surfactant, viscosity showed a slight increment. The behavior of Agar, characterized by an immediate increase in viscosity, restricted permeation into porous media, while CMC only slowed down the permeation process. The overall viscosity of the A1C1 final formulation increased over time due to the dominance of CMC’s rising viscosity over the thinning effect caused by Agar’s presence. Furthermore, the drilling fluid containing nano clay exhibited a lower viscosity drop compared to other formulations. Although the viscosity of the drilling fluid containing Agar at 150 °F remained stable, the viscosity of the base mud displayed unstable behavior. Moreover, the stability of the polymer viscosity significantly influenced the stability of the A1C1 compound at a shear rate of 1022 s^−1^.

In micromodel experiments, initially, the base drilling fluid caused the most formation damage, followed by the penetration of filtrate into the porous media. In highly permeable formations, a large amount of mud could enter the formation, where Agar bridging agent could be employed to prevent fluid loss. In contrast, CMC was unable to form a filter cake on the wellbore, resulting in a larger invaded zone. Therefore, better results could be achieved by reducing the content of CMC. It was observed that in the A1C1 formulation, CMC reduced the thickness of the filter cake. Finally, the mud cake formed in the A1C1 + nano1 wt% formulation exhibited minimal penetration and showed great potential for effectively sealing high permeable areas with a thin and smooth filter cake.

## Data Availability

All the data are available from the corresponding author on reasonable request.

## Abbreviations

API: American Petroleum Institute
IFT: Interfacial tension
C: Carboxymethyl cellulose (CMC)
A: Agar
S: Sodium dodecyl sulfate (SDS)
S004: 0.04% wt of SDS
C05: 0.5% wt of CMC
τ: Shear stress
τ_y_: Yield stress
$$\dot{\gamma }$$: Shear rate
m, m_b_: Consistency index
n: Power law coefficient
η_∞_: Viscosity at infinite shear rate
η_0_: Viscosity at zero shear rate
F: Fahrenheit
